# Effects of valproic acid, levetiracetam, carbamazepine, and oxcarbazepine on thyroid function tests in children

**DOI:** 10.1590/1806-9282.20241177

**Published:** 2024-12-16

**Authors:** Olcay Güngör, Beste Kipçak Yüzbaşı, Bayram Özhan, Onur Orhan, Rümeysa Şevik, Gülay Güngör

**Affiliations:** 1 Pamukkale University, School of Medicine, Department of Pediatric Neurology – Denizli, Turkey.; 2 Pamukkale University, School of Medicine, Department of Pediatric Endocrinology – Denizli, Turkey.; 3 Pamukkale University, School of Medicine, Department of Public Health – Denizli, Turkey.; 4 Pamukkale University, Faculty of Medicine, Department of Pediatrics – Denizli, Turkey.; 5 Pamukkale University, School of Medicine, Department of Radiology – Denizli, Turkey.

**Keywords:** Anticonvulsants, Children, Thyroid hormone

## Abstract

**OBJECTIVE:**

The aim of the study was to evaluate the effects of commonly used medications for epilepsy on thyroid function tests in children.

**METHODS:**

Epileptic children treated with valproic acid, levetiracetam, carbamazepine, and oxcarbazepine were retrospectively examined along with a healthy control group. Levels of free thyroxine 4 and thyroid-stimulating hormone were compared.

**RESULTS:**

In patients receiving valproic acid monotherapy, thyroid-stimulating hormone levels increased compared to both the control group and pre-treatment levels, while free thyroxine 4 levels remained unchanged. In those receiving carbamazepine or oxcarbazepine monotherapy, average free thyroxine 4 levels were found to be lower compared to the control group and pre-treatment levels, with no difference in thyroid-stimulating hormone levels. For patients receiving levetiracetam monotherapy, there was no difference in free thyroxine 4 and thyroid-stimulating hormone levels compared to the control group and pre-treatment levels.

**CONCLUSION:**

Despite significant changes in thyroid hormone levels with valproic acid, carbamazepine, and oxcarbazepine treatment, no significant clinical findings were observed. Additionally, no effect of levetiracetam on thyroid function tests was detected.

## INTRODUCTION

Epilepsy is a common neurological disorder in childhood and is usually treated with antiepileptic drugs (AEDs). AEDs can be associated with certain side effects, one of the lesser-known being subclinical hypothyroidism. Subclinical hypothyroidism is characterized by an increase in serum thyroid-stimulating hormone (TSH) levels, while fT4 levels remain within the normal range. Antiepileptic drugs can affect thyroid function through various mechanisms. For instance, drugs such as valproate and carbamazepine can influence the peripheral conversion of thyroid hormones, the levels of thyroid hormone-binding globulin, and the functions of the thyroid gland. Specifically, carbamazepine and oxcarbazepine affect thyroid function in children by inducing hepatic enzymes, while valproic acid (VPA) impacts thyroid function through various mechanisms (e.g., gamma aminobutyric acid (GABA) effects, zinc deficiency, etc.). The effects of levetiracetam (LEV) on thyroid function remain unclear^1,2^. These drugs can cause an increase in serum TSH levels, which may lead to the development of subclinical hypothyroidism. Subclinical hypothyroidism is usually asymptomatic and is diagnosed through laboratory tests. Diagnosing subclinical hypothyroidism in children requires consideration of age-specific TSH and T4 reference ranges. In this study, we aimed to investigate the effects of LEV, VPA, carbamazepine (CBZ), and oxcarbazepine (OCX) monotherapy on thyroid hormones in children with epilepsy.

## METHODS

We retrospectively reviewed the records of patients aged 3–18 years who were seen at the Pediatric Neurology Clinic of Pamukkale University Medical School Hospital between January 2019 and 2024. The study included 237 patients who were on monotherapy: 75 on VPA, 80 on LEV, 48 on CBZ, and 34 on OXC, as well as 200 individuals in the control group. Idiopathic epileptic children who had been on VPA, LEV, CBZ, or OXC monotherapy for at least 12 months and had been seizure-free for at least 6 months were included in the study. Patients were treated with VPA monotherapy at doses ranging from 10 to 40 mg/kg/day, LEV at 20 to 45 mg/kg/day, CBZ at 10 to 20 mg/kg/day, or OXC at 10 to 35 mg/kg/day. The diagnosis of epilepsy was made according to the definition and classification of the International League Against Epilepsy (ILAE)^3^. All children had normal neurological examination and cranial imaging findings. Thyroid functions of patients on monotherapy and healthy children (control group) were compared at 0, 6, and 12 months. Serum VPA and CBZ levels were measured. LEV and OXC levels were not measured. The chemiluminescence immunoassay method was used to measure TSH and fT4 levels (Abbott Architect i200SR, USA). Laboratory reference values were considered normal, with fT4 levels between 0.7 and 1.5 ng/dL and TSH levels between 0.5 and 5 uIU/mL. Subclinical hypothyroidism was characterized by a TSH level exceeding 5 uIU/mL while maintaining a normal fT4 level^4^. Patients with chronic disease, mental retardation, and those using multiple AEDs were excluded from the study. Ethical approval was obtained.

### Statıstıcal analysıs

Data were analyzed using the Statistical Package for the Social Sciences (SPSS) software (version 13.0). One-way ANOVA was used for comparing means across multiple groups, with Turkey and Tamhane tests for multiple comparisons. Pearson chi-square and Fisher exact tests were used for categorical variables. Repeated measures analyses with general linear models were conducted for treatment measurements at baseline, 6 months, and 12 months, and between patient groups. The Bonferroni test was applied for paired comparisons along with Pillai's Trace test. Pearson and Spearman correlations assessed relationships within patient groups. A p<0.05 was considered significant.

## RESULTS

The study included 241 children with epilepsy and 200 children in the control group. Demographic information is provided in Table 1. There was no difference in age and gender when the patient and control groups were compared. At the initial evaluation, thyroid hormone levels were within normal ranges for both the epileptic children and the control group. The baseline hormone levels did not show any statistically significant variation across the groups (p>0.05). In patients taking VPA, TSH levels were markedly elevated at both the 6-month and 12-month marks compared to the control group and baseline measurements (p<0.001), while fT4 levels remained unchanged (p>0.05). In patients on CBZ and OXC, fT4 levels were lower at the 6th and 12th months compared to the control group and baseline values (p<0.001), but they remained within the normal reference range. TSH levels did not change (p>0.05). In patients treated with LEV, fT4 and TSH levels remained stable at both 6 and 12 months compared to the control group and initial measurements (p>0.05) (see Table 2). Before treatment, there were no significant differences in thyroid hormone levels and body mass index (BMI) between the control group and the medication groups. Subclinical hypothyroidism was observed in 12 patients (16%) from the VPA group, 2 patients (1%) from the CBZ group, and 1 patient (0.7%) from the OXC group. No hypothyroidism was detected in the LEV group. There were no clinical symptoms that would suggest hypothyroidism in our cases. There was no relationship found between the dosage or length of medication and the levels of fT4 and TSH.

**Table 1:** Patient characteristics.

	Controls	VPA	CBZ	OXC	LEV	p
Year	8.10±2.63	8.12±2.53	7.10±2.74	7.23±2.13	9.21±2.43	p0>0.05
Gender (n)	200	75	48	34	80	
Male/female (n)	110/90	40/35	26/22	19/15	37/43	p0>0.05

**Table 2:** Changes in serum hormone levels during and after treatment with antiepileptic.

VPA	Control group	Before treatment	6th month of treatment	12th month of treatment	p-value
fT4	1.27±0.24	1.26±0.17	1.26±0.17	1.27±0.15	p0>0.05 p1>0.05 p2>0.05 p3>0.05 p4>0.05
TSH	2.38±1.3	2.41±1.3	2.74±1.1	2.81±1.2*	p0>0.05 p1<0.01* p2<0.001* p3<0.001* p4<0.001*
**CBZ**	**Control group**	**Before treatment**	**6th month of treatment**	**12th month of treatment**	**p-value**
fT4	1.20± 0.24	1.19±0.22	1.14±0.21	1.13*±0.23	p0>0.05 p1<0.01* p2<0.001* p3<0.001* p4<0.001*
TSH	2.45±1.24	2.49±1.24	2.471±1.17	2.44±1.20	p0>0.05 p1>0.05 p2>0.05 p3>0.05 p4>0.05
**OXC**	**Control group**	**Before treatment**	**6th month of treatment**	**12th month of treatment**	**p-value**
fT4	1.23±0.24	1.24±0.24	1.17±0.23	1.14±0.20*	p0>0.05 p1<0.01* p2<0.001* p3<0.001* p4<0.001*
TSH	2.19±1.24	2.20±1.23	2.23±1.21	2.24±1.31	p0>0.05 p1>0.05 p2>0.05 p3>0.05 p4>0.05
**LEV**	**Control group**	**Before treatment**	**6th month of treatment**	**12th month of treatment**	**p-value**
fT4	1.18	1.19±0.18	1.17±0.17	1.16±0.13	p0>0.05 p1>0.05 p2>0.05 p3>0.05 p4>0.05
TSH	2.05±1.36	2.10±1.36	2.12±1.28	2.11±1.31	p0>0.05 p1>0.05 p2>0.05 p3>0.05 p4>0.05

P0: baseline levels compared with the control group; P1: 6-month levels compared with the baseline; P2: 6-month levels compared with the control group; P3: 12-month levels compared with the baseline; P4: 12-month levels compared with the control group; TSH: thyroid-stimulating hormone; fT4: free thyroxine; VPA: valproic acid; CBZ: carbamazepine; OXC: oxcarbazepine; LEV: levetiracetam. Values are expressed as mean (standard deviation).

* Statistically significant value.

## DISCUSSION

We observed thyroid hormone abnormalities in epileptic patients using VPA, CBZ, and OXC, but not in those using LEV. This suggests that some antiepileptic drugs may affect thyroid function. The relationship between thyroid hormones and epilepsy is complex, with some suggesting epilepsy or AEDs might impact thyroid function^1,2^. Since thyroid hormone levels were normal before treatment and after the first convulsions, we assume that the observed changes were not due to the convulsive disorder itself.

In our patients using VPA therapy, despite an increase in TSH levels compared to pre-treatment and control groups, there was no change in fT4 levels, and they were clinically euthyroid. A meta-analysis reported significantly higher TSH levels in 12–52% of patients on VPA monotherapy^2^. In a study of 45 cases over 6 months, TSH increased, fT4 decreased, and fT3 increased^5^. However, few studies have reported no changes in thyroid function tests^6,7^, and severe hypothyroidism is rare^8^. Our study found subclinical hypothyroidism in 18% of children on VPA therapy, consistent with Karatoprak et al. (16%)^9^ and Alhyan et al. (17.7%)^5^. The literature indicates that changes in thyroid hormone levels with valproic acid are not permanent and normalize after discontinuation of the drug^2,8^.

There are multiple mechanisms through which valproic acid affects thyroid hormones. Valproic acid can increase TSH levels due to its effects on GABA. It inhibits somatostatin, which suppresses TSH secretion; it can cause zinc and selenium deficiencies, disrupting thyroid hormone synthesis; and it can lead to magnesium deficiency, which reduces iodine uptake and T4 synthesis, resulting in increased TSH secretion. Additionally, magnesium deficiency can impair the effects of thyroid hormones^2^.

In our study, although there was a reduction in serum fT4 levels at both 6 and 12 months in patients receiving CBZ compared to the control group and baseline levels, these levels remained within the normal reference range. We found no change in TSH levels. Our findings are consistent with a previous meta-analysis that reported an association between CBZ use and low serum fT4 levels without changes in TSH levels in children^10^. According to the literature, 28–33% of patients using CBZ had a reduction in fT4 levels^2^. Most studies have found decreases in T4, fT4, T3, and fT3 even after just 1 month of treatment^2,11–13^. Some studies also reported elevated TSH levels in 10–25% of patients after 12 months of treatment^2,13^.

Another study reported a significant decrease in serum fT4 at 1 and 6 months in eight CBZ-treated cases, with no change in TSH levels^11^. The strengths of this study are its longer follow-up and larger sample size. The clinical significance of low serum thyroid hormone levels remains unclear, and long-term, multicenter studies are needed to assess the impact on asymptomatic individuals.

The decrease in serum thyroid hormones may be due to the hepatic P450 enzyme complex of carbamazepine, which accelerates the metabolism of thyroid hormones. The positive feedback mechanism of the hypothalamic-pituitary-thyroid axis may not respond to these decreases in fT4 and fT3 levels, and TSH levels may remain normal. Carbamazepine's blocking of thyroid hormone binding to thyroxine-binding globulin and increasing peripheral conversion of triiodothyronine lead to lower fT4 levels compared to the control group^1,14^. However, the clinical significance of low serum thyroid hormones is unclear. Long-term, multicenter studies are needed to determine the impact of these changes in asymptomatic individuals.

In our study, despite a decrease in serum fT4 levels in children treated with OXC at 6 and 12 months compared to the control group and pre-treatment levels, the values remained within the normal reference range. We found no change in TSH levels. Both retrospective and prospective studies have shown that patients using oxcarbazepine often experience decreased serum T4 and fT4 levels without changes in TSH levels^13,15–18^. This suggests that OXC can alter thyroid function in epileptic children within a short period. Unlike carbamazepine, oxcarbazepine does not induce similar enzyme activity despite its structural similarity. fT4 levels normalize when oxcarbazepine is discontinued. As a result, the effect of oxcarbazepine on thyroid function is not permanent and is well tolerated compared to carbamazepine^13^. Our data show that although OXC affects thyroid hormone levels, it does not cause clinical findings.

synaptic vesicle glycoprotein 2A (SV2A), present in the central nervous system (CNS) and endocrine tissues, is the target of LEV, suggesting that it might influence endocrine functions^19^. However, our study found no changes in thyroid hormone levels with LEV monotherapy over 6 and 12 months compared to controls and pre-treatment values. These findings align with other studies, both short and long terms, and may be due to LEV's unique antiepileptic mechanism^9,11,15,20^. The lack of thyroid hormone abnormalities in many studies could be related to LEV's distinct mode of action.

The relationship between thyroid hormone levels, duration, or dose of treatment is unclear; some studies found a relationship between doses of OXC or VPA and TSH levels^21,22^, while others did not^14^. Our study found no relationship between the daily doses of VPA and CBZ and the levels of TSH. The limitations of the study include its retrospective design, the inability to assess the impact on thyroid function after discontinuation of treatment, and the small sample sizes in the OXC and CBZ groups.

## CONCLUSION

It should be noted that valproic acid does not affect thyroid hormones significantly, but subclinical hypothyroidism may develop, necessitating close monitoring of symptoms. Although CBZ leads to significant changes in thyroid hormone levels, these changes do not appear to cause clinical symptoms. Routine monitoring of thyroid hormone levels in patients on LEV is generally not required. Longer-term studies are needed to better understand antiepileptic treatment's impact on thyroid function.
